# Survey of Australians using cannabis for medical purposes

**DOI:** 10.1186/1477-7517-2-18

**Published:** 2005-10-04

**Authors:** Wendy Swift, Peter Gates, Paul Dillon

**Affiliations:** 1 National Drug and Alcohol Research Centre, University of New South Wales, Sydney, NSW, 2052 Australia

## Abstract

**Background:**

The New South Wales State Government recently proposed a trial of the medical use of cannabis. Australians who currently use cannabis medicinally do so illegally and without assurances of quality control. Given the dearth of local information on this issue, this study explored the experiences of medical cannabis users.

**Methods:**

Australian adults who had used cannabis for medical purposes were recruited using media stories. A total of 147 respondents were screened by phone and anonymous questionnaires were mailed, to be returned by postage paid envelope.

**Results:**

Data were available for 128 participants. Long term and regular medical cannabis use was frequently reported for multiple medical conditions including chronic pain (57%), depression (56%), arthritis (35%), persistent nausea (27%) and weight loss (26%). Cannabis was perceived to provide "great relief" overall (86%), and substantial relief of specific symptoms such as pain, nausea and insomnia. It was also typically perceived as superior to other medications in terms of undesirable effects, and the extent of relief provided. However, nearly one half (41%) experienced conditions or symptoms that were not helped by its use. The most prevalent concerns related to its illegality. Participants reported strong support for their use from clinicians and family. There was almost universal interest (89%) in participating in a clinical trial of medical cannabis, and strong support (79%) for investigating alternative delivery methods.

**Conclusion:**

Australian medical cannabis users are risking legal ramifications, but consistent with users elsewhere, claim moderate to substantial benefits from its use in the management of their medical condition. In addition to strong public support, medical cannabis users show strong interest in clinical cannabis research, including the investigation of alternative delivery methods.

## Background

While cannabis has long been part of folk pharmacopeia, there is a burgeoning body of research on its therapeutic potential. This has largely drawn on scientific advances in our understanding of the pharmacology of cannabis, and its complex interactions with the central nervous system, particularly endogenous brain reward pathways [1]. In addition to basic experimental research, case reports, surveys of people using cannabis for medical conditions and prospective clinical trials of cannabis-based medicines are consolidating the evidence that cannabis may play a role in the management of some medical conditions. Authoritative reviews of this evidence indicate that cannabis has therapeutic potential for conditions such as HIV- and cancer-related wasting, nausea and vomiting resulting from chemotherapy, neurological disorders such as multiple sclerosis and chronic pain [1-4].

While current research reveals exciting therapeutic opportunities, there is an ongoing debate about the virtues of obtaining such benefits from the complex chemical cocktail contained in the whole plant or from one or more components isolated and developed into a synthetic pharmaceutical product. This debate cross-cuts important issues such as the difficulties of reliable dosing when using the natural product, whether the potential harms of smoking cannabis due to its ease of titration overshadow its therapeutic benefits, and whether different medical conditions will respond more favourably to the whole plant or to different constituents in isolation or combination. However, underlying these issues is the reality that most people who use cannabis medicinally do so by using black market supplies of an illicit drug.

As with the opiates, evaluations of the therapeutic potential of cannabis occur in the context of a vigorous political debate on the use of an illicit drug with dependence potential for medicinal purposes. This situation is clearly evident in the United States, where there is an ongoing legal challenge by the Federal Government over the States' rights to allow cannabis to be used by registered medical users. Despite Canada's recent decision to provide a controlled supply of natural cannabis to registered users, and approvals for the marketing of Sativex, a pharmaceutical cannabis extract, in some countries, currently most users would rely on home-grown cannabis, or supplies obtained from friends, families, dealers and medical compassion clubs.

To date, there has been little interest in Australia in formally investigating the therapeutic potential of cannabis or investigating the practices of current medical users. In 1999 the NSW State Government commissioned a Working Party to investigate the issue and recommend research and legislative options. Among their recommendations were: controlled clinical trials of cannabis, investigations into delivery methods other than smoking, surveys of current medical cannabis users and legislative amendments to allow compassionate use [4]. Subsequently, in 2003 the NSW Government announced it would conduct clinical trials, but despite generating significant publicity, there has been no further commitment by the NSW Government on this issue. The 2004 National Drug Strategy Household Survey found widespread public support for medical cannabis use, with 68% supporting a change in legislation to permit use for medical purposes and 74% supporting a clinical trial of medicinal cannabis use [5]. It is not known how many people use cannabis for medicinal purposes in Australia. Those who do use it engage in an illegal behaviour and risk arrest. Those that rely on black market supplies use a product of unknown source and quality.

Several surveys in the US, UK, Germany and Canada [6-12] have reported perceived improvements in a variety of medical conditions following cannabis use. However, we know very little about the experiences of Australian users, and how they compare to findings in other studies. These authors are aware of only two unpublished Australian studies conducted in northern NSW; in 1998 a survey of 202 users recruited at the Nimbin HEMP Embassy [13], and in 2003 a survey of 48 members of a medical cannabis information service [14].

This paper presents the results of a study of 128 users, which aimed to learn more about their patterns of use, experiences and concerns, and interest in participating in a medical cannabis trial.

## Methods

### Sample

The sample comprised 128 people who used cannabis for medical purposes. To be eligible for the study, participants had to be living in Australia and to be currently using/have previously used cannabis for medical purposes. While the study targeted residents of Australia's most populous state, NSW (pop: approximately 6.7 million), we did not exclude participants from other parts of Australia (total pop: approximately 20 million).

As it is not known how many Australians use cannabis for medical purposes it was not possible to obtain a representative sample of such users. As this was an exploratory study to see who responded to a general call for participation in the survey, we did not target groups representing people with specific medical conditions (e.g., HIV/AIDS, multiple sclerosis) or hospital departments known to treat patients who may benefit (e.g., oncology, chronic pain clinics). Participants were primarily recruited from opportunistic media stories between November 2003 and August 2004, in newspapers, on radio and television. In addition, the Medical Cannabis Information Service (MCIS) in Nimbin, NSW, offered to tell its members about the survey and the International Association for Cannabis as Medicine (IACM), in Germany, placed the questionnaire on its website.

A total of 147 enquiries were received between December 2003 and August 2004 by telephone and email and approximately 170 questionnaires distributed (some people requested multiple copies to distribute). For example, the media stories generated enquiries from several GPs who said they would inform certain patients of the study. Of the 131 questionnaires returned, 128 were used for analysis (75% of questionnaires sent out). Of the three discarded questionnaires, one respondent was a recreational cannabis user and two had never used cannabis.

### Questionnaire

The survey comprised an anonymous mail-out questionnaire, adapted from one developed by the MCIS in a recent study of its members [14]. Several issues were covered, including medical conditions/symptoms experienced, patterns of medical cannabis use, symptom relief and effects of use, comparison of cannabis to other medications, source and legal concerns (e.g., arrest), other concerns over use, opinion of family, friends and medical personnel, and interest in participating in a cannabis trial. The final version incorporated comments from researchers and clinicians interested in this issue.

### Procedure

The study received ethics approval from the University of New South Wales Social/Health Human Research Ethics Advisory (HREA) Panel. Interested persons were screened for eligibility over the phone and informed of the purpose of the survey; assurances of anonymity and confidentiality were provided. Questionnaires were mailed to participants, completed anonymously and returned in a stamped, self-addressed envelope. Addresses were destroyed when the questionnaire was posted.

### Analyses

Data were entered into SPSS (Version 12.0.1). As this was an exploratory study with a small sample size, this paper reports descriptive statistics only. Percentages are presented for categorical data; means (for normally distributed) and medians (for skewed data) are presented for continuous data. While data are usually presented on the overall sample, gender and age differences are presented for some variables, where they are of interest.

## Results

### Demographics

The sample was 63% male. Participants had a median age of 45 yrs (range 24–88), with almost one third (31%) aged 50 years or over, and one in ten (9%) aged 60 years plus. While the study targeted NSW residents (who represented 58% of participants), responses came from across Australia, especially Queensland (15%) and Victoria (12%). Residents of other States and Territories each comprised less than 3% of participants.

Participants reported a wide range of medical conditions and symptoms associated in the literature with the use of medicinal cannabis (Table 1), most commonly chronic pain (53%) and arthritis (38%). Approximately one in five reported migraine (22%), weight loss (21%) and persistent nausea (20%). However, depression was the most commonly reported condition/symptom (60%). Up to 35 other conditions/symptoms were listed, most commonly post traumatic stress disorder (PTSD) (5%) and irritable bowel syndrome (4%). It is important to note that we did not ask participants to distinguish between primary symptoms/conditions for which they sought treatment (e.g., cancer) and conditions which may have been secondary to this (e.g., depression) or consequent to treatment (e.g., chronic nausea). Multiple conditions (mean = 3.7, SD = 2.1, range = 1–10), of lengthy duration, were the norm, with three quarters (84%) reporting more than one condition and two thirds (67%) at least three conditions. Congruent with this picture, cannabis was used to relieve multiple symptoms (median = 3, range = 1–12), especially chronic pain (57%), depression (56%), arthritis (35%), persistent nausea (27%) and weight loss (26%).

**Table 1:** Conditions/symptoms experienced, duration, and conditions/symptoms requiring cannabis relief (n = 128).

Condition	(%) with condition	Median duration (yrs)	% used cannabis for relief of..*
Depression	60	10	56
Chronic pain	53	10	57
Arthritis	38	9	35
Migraine	22	18	17
Weight loss	21	4	26
Persistent nausea	20	6	27
Spinal cord injury	14	11	13
Spasms (spasticity)	13	8	16
Fibromyalgia	13	13	13
Wasting	13	5	11
ME (chronic fatigue)	13	16	13
Neuralgia/neuropathy	12	8	12
HIV/AIDS	9	15	8
Multiple sclerosis	7	9	7
Cancer	6	10	4
Other neurological disorder	6	5	6
PTSD	5	13	1 person
Irritable bowel syndrome	4	10	1 person
Glaucoma	3	29	2

*These figures do not necessarily equate with the % reporting a particular condition because some people reported using cannabis to relieve the particular symptoms (e.g., chronic pain, nausea) associated with a condition, rather than citing they used cannabis to relieve the condition itself (e.g., arthritis, cancer).

### Patterns of medical cannabis use

Participants had first tried cannabis for medical purposes at a median age of 31 years (range = 14–77). More than one quarter (29%) had discovered its therapeutic benefits as a spin-off from recreational use; others had tried it following concerns about the side-effects of their medications (14%), or a belief their medications or treatment were ineffective (13%), or had acted on the recommendation of a medical practitioner (10%) or friend (10%).

Table 2 presents data on patterns of medical use. Most (85%) were currently using cannabis therapeutically, even if sporadically. For those who had stopped, the main reasons were: their inability to obtain a regular supply (9/19 people), its illegality (7/19), cost (7/19) and disliking the side effects or route of use (each 3/19). Of those using intermittently, many reported their use would be more regular if it were more readily availability and cheaper.

**Table 2:** Patterns of medical cannabis use (n = 128 unless specified)

	Total (%)	Male (%)	Female (%)
Current use	85	86	83

Length of use			
<1 year	12	9	17
1–5 yrs	27	23	35
6–10 yrs	20	26	10
11–15 yrs	9	10	8
16–20 yrs	10	10	10
>20 yrs	21	23	19

Frequency of use (n = 126)			
several times a day	39	45	29
6–7 days/wk	24	19	31
1–5 days/wk	14	14	13
less than weekly	2	3	2
very seldom	2	1	2
as required	20	18	23

Method(s) of use (n = 127)			
eaten as cooked recipe	49	48	50
drunk as tea	7	8	6
smoked as cigarette (joint)	65	58	77
smoked as dry pipe (chillum)	24	28	19
smoked as water pipe (bong)	54	58	46
vaporiser	8	11	2
eaten as leaf/flower matter	3	4	2

Most helpful method of use (n = 126)			
eaten as cooked recipe	16	15	17
drunk as tea	2	3	2
smoked as cigarette (joint)	31	26	40
smoked as dry pipe (chillum)	10	13	4
smoked as water pipe (bong)	33	36	29
vaporiser	2	3	2
other	6	5	6

Medical use was typically long-term and regular. Use of less than one year was uncommon (12%), with more than half (61%) having used it for at least six years; one in five reported very long-term use (more than 20 years). Most used at least weekly (75%), and more than half (59%) used almost daily or daily. Approximately one in five (22%) specified they used it "as required" for their condition (e.g., when pain was severe). Women tended to report shorter term use than men (52% vs. 31% citing use of 5 years or less).

It was most common for participants' medical use to be stable (22%) or largely unchanged since they started (17%), although it was most common for the amount used to vary according to their condition (35%). About one in ten indicated some increase in dose had been required (12%), while few reported a decrease (5%). Women tended to report more variable (44% vs. 29% of men) or short term use (15% vs. 6% of men); men tended to report an increase in the amount needed (17% vs. 4% of women).

In addition to medical use, three quarters (80%) of participants had used cannabis recreationally. Recreational use was less common among older participants (75% and 97% of recreational users were aged less than 50 years and 65 years, respectively). For almost half (46%), use in the past year had been solely medicinal, but the remainder reported recent recreational use – 29% in the past week, 19% in the past month and a further 6% in the past year.

### Route of use

While most people had tried multiple routes for relief, overall smoking was the route most commonly reported (91%). Approximately half the sample (49%) also smoked tobacco, and two thirds (64.1%) mixed their cannabis with tobacco.

Eating cannabis in cooked recipes was also very prevalent (49%). While vaporisers are not readily available in Australia, 8% had used them. In addition, four people had used tinctures and one used it topically in the bath or as a cream for a skin condition. Overall, smoking was also considered to be the most *helpful *route of use for symptom relief (74%), although concerns about this route of use were widespread. Consistent with Australian research on preferred route of use and age [15], older users (aged 50 years +) typically found joints the most helpful method of use (41% vs. 26% of younger users), while younger users preferred the use of waterpipes (43% vs. 13% of older users).

When asked to comment on the good and bad points of different methods of ingestion the most consistent response was that smoking of any form, particularly with tobacco, was detrimental to respiratory function (and health). This was of particular concern to non-smokers, some of whom did not know how to cook cannabis recipes. Despite attracting the bulk of negative comments, its popularity seemed to lie with its instant effect, its ease of titration and cost-effectiveness compared to the oral route. It seemed to "do the job". Eating was seen to be a much healthier option – it was "safer", tasty when cooked in a recipe, less obvious than smoking and could be done virtually anywhere. Some people liked its slow onset and long-lasting effects, but others claimed difficulties with titration and slow onset made it expensive and ineffective for rapid symptom relief.

### Effects of cannabis use

When asked to rate the overall effects of cannabis on a Likert scale ranging from "I feel a lot worse" to "gives me great relief", cannabis was perceived to provide "great relief" (86%) or a little relief (14%). No one believed it had been detrimental to their condition or symptoms.

Positive ratings were ("great" or "good" relief) were also typical for its ability to relieve specific symptoms (Table 3). In addition, several other symptoms were noted, primarily insomnia (13% used for insomnia; of these 82% derived "great" relief).

**Table 3:** Symptom relief (n = 128)

Symptom relief required...*	Total (%)	Male (%)	Female (%)
Nausea relief	48	56	44
Of these, received:			
great relief	53	51	62
good relief	44	46	35
no effect	3	3	4

Pain relief	83	83	83
Of these, received:			
great relief	55	49	65
good relief	45	52	35
no effect	0	0	0

Ability to cope emotionally	66	70	60
Of these, received:			
great relief	45	40	54
good relief	54	58	46
no effect	1	2	0

Appetite stimulant	51	55	44
Of these, received:			
great relief	52	55	48
good relief	46	46	48
no effect	2	0	5

Decrease in spasms/tremor	39	36	44
Of these, received:			
great relief	43	43	43
good relief	55	54	57
no effect	2	4	0

Relief through relaxation	83	88	75
Of these, received:			
great relief	72	69	78
good relief	28	31	22
no effect	0	0	0

* No-one reported their condition was made worse

Approximately three quarters of participants (71%) claimed to have experienced a return of their symptoms or condition on stopping cannabis, especially: pain (53% of those who claimed a return of symptoms), depression or anxiety (30%), insomnia (11%), spasm (10%) and nausea/vomiting or lack of appetite (9%).

Only one in ten (11%) participants reported symptoms they believed were unrelated to their medical condition upon stopping cannabis, citing symptoms congruent with cannabis withdrawal such as anxiety or mood disturbance (including paranoia), insomnia, loss of appetite, restlessness and vivid dreams.

### Comparison with other medicines

Almost two thirds (62%) of respondents claimed that they decreased or discontinued their use of other medicines when they started using cannabis medicinally. This was more common in males (65% vs. 58% of females) and older participants (aged 50 years +) (70% vs. 59% among younger participants). For some people this was a substantial change, representing a shift away from chronic, high-dose medication use.

Perhaps not surprisingly, cannabis was typically perceived as superior to other medications in terms of undesirable effects, and the extent of relief provided (Table 4). Thus, cannabis was rated to produce equivalent (8%) or worse side effects (3%) by a minority of therapeutic users. It was considered to work "a bit" or "much better" than other medicines, or to be the only source of relief, by more than three quarters (82%). Two participants made the interesting comment that cannabis worked *differently *to other medicines, so could not be directly compared.

**Table 4:** Comparison of cannabis with other medications (n = 128 unless specified).

	Total	Male	Female
Decreased or discontinued use of other medicines (n = 117*)	62%	65	58

Comparison of undesirable effects (n = 125)			
Cannabis produced much worse effects than other medicines	1	0	2
Cannabis produced somewhat worse effects	2	4	0
Undesired effects about the same	8	8	9
Other meds produced somewhat worse effects than cannabis	16	14	19
Other medicines produced much worse effects than cannabis	41	40	43
I have no undesirable effects from cannabis	31	33	28
			
Other medicines work differently	1	1	0

Comparison of relief provided (n = 118*)			
Other medicines work much better than cannabis	3	0	7
Other medicines work a bit better than cannabis	3	4	0
Other medicines work about the same as cannabis	9	8	9
Cannabis works a bit better than other medicines	13	11	15
Cannabis works much better than others medication	54	58	48
Only cannabis gives me relief from my condition	15	15	15
			
Other medicines work differently	2	0	4
Can't distinguish – use them together	1	1	2
Use cannabis to relieve side effects of other medicines	1	1	0

*Some people did not use other medications concurrently

Despite the very positive response to the use of cannabis, nearly one half (41%; 36% of men and 50% of women) found it did not help certain conditions/symptoms. Almost one third (29%) said cannabis was less effective for certain types of pain, or extreme pain, with a further 12% specifying migraine or headache pain. Nearly one in ten (8%) reported no effect on depression or anxiety. More than one in ten (14%) specified that while cannabis could ease their symptoms and enabled them to cope, they realised that it could not cure their underlying condition. Younger participants were more likely than older participants to claim a condition not helped by cannabis (45% vs. 32% of those aged 50 years +).

### Supply issues

Participants obtained medical cannabis from multiple sources (median = 1, range = 1–6; 44% had two or more sources), especially friends or family (58%) and dealers (42%). A substantial proportion grew their own (38%) while few (6%) obtained it from a compassion club or cooperative. Among those who purchased cannabis, the median weekly outlay was $50 (range = $1–$500, n = 95).

When asked to comment on the variability of the cannabis they used, those who could obtain a consistent supply of high quality cannabis that suited their needs were in the minority. Typically, participants noticed variability along a number of lines, such as potency, effectiveness, intoxication and side-effects, which made titration difficult. While some noted the importance of factors such as the part of the plant used (e.g., leaf versus head/buds), strain (e.g., *sativa *versus *indica*), soil and climate, the overwhelming responses focussed on hydroponic versus soil-grown cannabis ("bush bud" or home grown cannabis), and home grown cannabis versus purchased cannabis.

Hydroponic cannabis was almost universally unpopular and was avoided where possible – despite its greater potency, it was also considered shorter acting, produced greater tolerance and worse side-effects than other cannabis. By comparison, soil-grown cannabis was perceived to be less unpleasantly potent, natural ("organic"), less chemically treated, and with fewer side-effects. However, it was also perceived as harder to get. Home grown cannabis was seen as the best method of obtaining a consistent, safe supply of medicinal quality. A common response was that purchased cannabis was not to be trusted, and that unscrupulous growers who were more concerned with yield and greed compromised the quality of their crop with chemicals such as growth hormone and pesticides.

### Concerns

A minority (13%) had no concerns over their medical cannabis use. Concerns over potential health effects (32%) or the risk of dependence (21%) were overshadowed by those relating to its illegal status (76%), the fear of being arrested (60%) and cost (51%). Indeed, one quarter (27%) claimed to have been arrested, cautioned or convicted in relation to their medical cannabis use, with this outcome more commonly reported by men (31% vs. 19% of women) and younger users (30% vs. 16% of users aged 50 years +). Other concerns mentioned (15%) were: the stigma of using, issues around parenting, pregnancy and relationships, availability, quality and difficulties in dose adjustment.

### Support from others and interest in clinical trial

Most participants had a regular doctor (90%) and about a half had a regular specialist (55%). Virtually all (90%) had informed a clinician of their therapeutic use, typically reporting a supportive response from GPs (75% of those told), specialists (74%) and nurses (81%). Family and friends were largely considered supportive of the participant's use (71%).

Not surprisingly, there was widespread support for Government provision of cannabis to patients in a variety of circumstances. At least three quarters supported the supply of cannabis to any patient who was permitted to use it by being registered under a Government scheme (82%); more specifically, those patients who: could not afford to buy it on a regular basis (82%), could only purchase it on the black market (81%), couldn't ensure a consistent supply (75%), or were worried about quality control issues (77%). More than half endorsed the supply of patients who did not know anyone capable of growing it (72%), were concerned about hydroponically grown cannabis (72%), or who needed a supply quickly (66%).

Although not all participants were NSW residents, there was almost universal interest (89%) in participating in a clinical trial, in which a controlled supply of cannabis was grown and provided to registered medical cannabis users. There was also strong, although lesser, interest in trying alternative delivery methods such as a spray or tablet (79%).

While for some people, the availability of any cannabis-derived product that worked was their prime concern, alternative delivery methods were considered attractive as they obviated the necessity to smoke, removed concern about engaging in illegal behaviour and having to access the black market, and were more portable and acceptable than smoking. The main caveats on an alternative were that it was easy to titrate, quick, efficient, reliable and natural or safe – sprays and vaporisers were mentioned specifically by some as preferable to pills in this regard. A clear theme was the desire to keep the holistic, natural properties of cannabis rather than produce a chemical/synthetic drug with numerous binding and carrying agents. Nevertheless, there was recognition that different medical conditions may require different approaches, such as different active agents (e.g., THC versus other cannabinoids), strains or methods (e.g., slow release pill versus fast-acting spray).

The main reason for not supporting alternatives appeared to be that using the whole plant in its natural state was perceived to be the best method. In addition, for some the ritual of cannabis use was perceived as part of its medicinal benefit. There was also concern at political interference and its potential for exploitation and corruption in a trial.

## Discussion

This exploratory study examined the patterns of medicinal cannabis use among a sample of 128 Australian adults who responded to media stories about this issue. Firstly, we need to acknowledge its limitations. As we do not know how many Australians use cannabis medicinally or their characteristics, we relied on the recruitment of volunteers through purposive sampling. Instead of targeting a particular group we used media stories disseminated widely on the radio, television and in newspapers to attract a cross-section of people. Thus, these results may not be representative of the experiences of all medicinal users, and may be affected by selection bias by excluding those who did not have access to these media, who did not wish to or could not contact us or did not return the questionnaire. We also attracted participants whose experiences with medical cannabis were typically positive, so they have little to tell us about people who have not found cannabis helpful or pleasant therapeutically. However, they still provide important information on these people's experiences, and raise important issues regarding the use of black market supplies of the cannabis plant and the development of cannabis-based pharmaceuticals. As the questionnaire was self-completed, there was potential for misunderstanding of the questions. However, the wording was straightforward, contact details were provided in the event of misunderstanding, and the results were remarkably consistent across participants, which encourages us that the questions were understood. Despite being anonymous, several participants provided us with contact details in case further information was needed, and wrote additional comments about their experiences and attitudes. In addition, many of the findings are remarkably consistent with the findings of other local and international studies, as indicated below.

People in this study reported regular, ongoing medical use over quite long periods – with 61% using for more than five years and 20% reporting very long-term use of more than 20 years. However, as Ware and colleagues noted in their study of almost 1000 medical users [10], this was a group of chronically ill people with multiple long-standing conditions. The perceived need for alternative or additional symptom relief may reflect the fact that we recruited a sample of particularly entrenched medicinal cannabis users who were dissatisfied with conventional treatments, that medicinal cannabis use is more likely to considered an option by people who find conventional treatments and medications unsatisfactory, or that many had been exposed to its perceived medical benefits quite early due to their recreational use. Larger studies addressing a broad cross-section of users may better answer this question.

Consistent with the literature on the conditions for which cannabis has been indicated, chronic pain, arthritis, persistent nausea and weight loss were among the most common conditions for which cannabis relief was sought. However, depression was the most common condition: more than half (56%) used cannabis to relieve depression, and two thirds (66%) used it to cope emotionally, universally obtaining great or good relief. Other studies have also reported cannabis use for the relief of depression, although not at this level [8-10,14]. The relationship between depression and cannabis use is controversial, with recent literature indicating that cannabis use may be implicated in depression and suicidal thoughts and behaviours This would suggest that regular medicinal use may be contraindicated by *placing *people at risk of depression or self-harm. However, we do not know the type or aetiology of the depression cited by our participants. Many may have experienced depression and stress associated with their physical condition, which may have been alleviated along with any physical relief. The risk may also be greatest among heavy, younger users and those who may already be vulnerable to mental ill health due to their life circumstances [16-18]. Medical cannabis use patterns may not typically be regular enough to pose a great risk. Regardless, it is important that people considering the use of medical cannabis are aware of the risks of use [19]. A recent paper [20] has suggested that THC and cannabidiol, two major components of cannabis, may help alleviate bipolar disorder, recommending a pharmaceutical product would be a safer option than crude cannabis, in which the balance of components is variable.

Consistent with local and international research on people with a variety of medical conditions [8-12,14], most participants claimed moderate to substantial benefits from cannabis, both in terms of their overall condition and management of individual symptoms. It was typically considered more effective and less aversive than other medications in managing their condition(s), the symptoms of which commonly re-emerged upon stopping (71%). While their use was often complementary to other medications and treatment, 62% had decreased or discontinued use of other medications when they commenced medicinal cannabis use. Nevertheless, cannabis was not a panacea – it did not help all conditions, particularly certain types of pain, and there was recognition that while it substantially improved quality of life it was not a cure. This is not necessarily surprising, as overall well-being and specific symptoms have multiple causes and can be affected by several factors, and is borne out by recent controlled clinical trials, for example, on chronic pain [21].

As others have reported (e.g., [8-10] we also found that in addition to medical use, recreational use was common: most (80%) had used cannabis recreationally, with about one half (54%) of these reporting some recent use. Indeed, 29% had discovered its therapeutic potential through their recreational use. One participant raised the issue that part of the therapeutic effect for them was the ritual of use and the "high" experienced [6]. This demonstrates the difficulty of precisely identifying the therapeutic component when people are using the natural plant matter, and will continue to present a challenge for the development of cannabis pharmaceuticals. While some people may find the illegality, route of use and psychoactive effects of natural cannabis undesirable and prefer a manufactured pharmaceutical product, several in this survey claimed to prefer the holistic delivery of all the compounds present when using the natural plant. We need to know more about the effect of the different active chemicals on medical conditions and how their therapeutic potential is mediated by the context of use.

Nonetheless, this was not simply a sample of recreational users, especially as we attracted many older users who used exclusively for medical reasons (75% of those aged 50 years+). They did not fit the recreational user stereotype, were willing to take the risk of using an illicit drug, exposure to the illicit drug market and the possibility of arrest to gain symptom relief. Indeed, the most common concern over medicinal use was its illegality, fear of arrest and cost (all >50%). One quarter (27%) of participants had experienced legal ramifications due to their use. Several people commented that they had no alternative than using an illegal drug, claiming that other medicines with negative and toxic effects (e.g., opiates) were legally prescribed, and that if nothing else worked for them they had the right to access cannabis without fear or stigma. Several made pleas for medical cannabis use to be treated as a medical, rather than a legal, issue, as their health and quality of life were at stake.

Smoking was the most common method of use; in addition, many were tobacco smokers or mixed cannabis with tobacco. Given the similarities between cannabis and tobacco smoke this is of particular concern for people who are ill, especially those with compromised immune systems. Despite acknowledgement of the risks of smoking and concerns expressed over its effects, it was considered the most helpful route of use. While eating was perceived as much healthier, until satisfactory solutions are achieved on titration and dosing issues, smoking will no doubt continue to be a popular method of obtaining relief.

Cannabis dependence was a concern for one in five participants (21%). This study provided indirect evidence that participants were unlikely to experience withdrawal symptoms on ceasing medical use, but this was only a crude measure. While the risk of dependence is probably low when used medicinally, this risk needs to be weighed up with the other concerns of the patient – for example, it may be low on the list of concerns for those with terminal illness [19].

Finally, participants reported that family and friends were likely to know about and support their medical cannabis use. These data also indicate that the medical profession is encountering, and frequently supporting, patients who use cannabis for symptom relief. Given their central role in the management of illness, it is important that clinicians are educated about the effects of cannabis, in order to assist patients in making informed decisions about their treatment. There was also clearly great interest among participants in a clinical trial and scope to investigate methods of delivery that avoid the health concerns associated with smoking cannabis, keeping in mind that some participants were reluctant to use a pharmaceutical product. In addition to distrust of unscrupulous participants in the black market, some were also distrustful of Government's motives and role in therapeutic research. It is therefore vital that any clinical trials are conducted in a rigorous, independent manner.

## Conclusion

Overall, these findings are consistent with those of other surveys, in revealing the perceived effectiveness of cannabis for the relief of symptoms associated with several medical conditions. While a small study, it has several implications. Firstly, people are risking the use of an illicit drug for its perceived therapeutic effects, and in some cases being arrested. Secondly, they are informing their clinicians about their medical use and frequently receiving support, highlighting the importance of ensuring clinicians are informed about cannabis. Finally, in addition to strong public support, medical cannabis users show strong interest in clinical cannabis research, including the investigation of alternative delivery methods.

## Competing interests

The author(s) declare they have no competing interests.

## Authors' contributions

WS conceived the study, designed the methodology, adapted the questionnaire, cleaned and analysed the data and wrote the paper.

PG assisted in questionnaire adaptation, managed data collection, entered the data, assisted with preliminary data analyses and commented on the manuscript.

PD assisted in questionnaire adaptation, recruited participants and commented on the manuscript.

All authors read and approved the final manuscript.
